# Frailty & Depression: Mediators of Fear of Falling and Quality of Life in Older Adults

**DOI:** 10.1093/geroni/igaa057.3344

**Published:** 2020-12-16

**Authors:** Michelle McKay, Christina Whitehouse, Janell Mensinger

**Affiliations:** Villanova University, Villanova, Pennsylvania, United States

## Abstract

Fear of falling is a known predictor for decreased health-related quality of life (QoL) in older adults, including among high risk frail community-dwelling older adults with multiple comorbidities including depression. The study aimed to examine the sequential explanatory roles of frailty and depressive symptoms in the relationship between fear of falling (FoF) and QoL in a program for all-inclusive care for the elderly (PACE). This was a retrospective single cohort study that included 84 older adults in a PACE program located in the Northeastern United States. Participants were cognitively intact older adults ≥55 years (M=70.33; SD=6.46). FoF was assessed with the Falls Efficacy Scale-International; frailty was measured with the Edmonton Frail Scale; QoL was measured with the Short Form 12v2 which includes both physical and mental domains. Using the Process Macro (model 6) in SPSS, path models were constructed hypothesizing frailty and depressive symptoms as serial mediators of the relationship between FoF and QoL while controlling for race, gender, age and comorbidities. Frailty and depressive symptoms serially mediated the FoF and mental QoL relationship (Indirect Effect = -0.10; 95% CI= -0.19, -0.03). Serial mediation effects of frailty and depressive symptoms were not replicated for the association between FoF and physical QoL (Indirect Effect = 0.00; 95% CI= -0.04, 0.05). Understanding the roles of frailty and depressive symptoms in explaining the association between FoF and mental health-related QoL can delineate targeted areas for intervention development not typically considered when attempting to reduce the influence of FoF on QoL in older adults.

